# Intermediate snowpack melt-out dates guarantee the highest seasonal grasslands greening in the Pyrenees

**DOI:** 10.1038/s41598-022-22391-x

**Published:** 2022-10-31

**Authors:** J. Revuelto, D. Gómez, E. Alonso-González, I. Vidaller, F. Rojas-Heredia, C. Deschamps-Berger, J. García-Jiménez, G. Rodríguez-López, J. Sobrino, R. Montorio, F. Perez-Cabello, J. I. López-Moreno

**Affiliations:** 1 Instituto Pirenaico de Ecología, Consejo Superior de Investigaciones Científicas (IPE-CSIC), Zaragoza, Spain; 2 Centre d’Etudes Spatiales de la Biosphère, CESBIO, Univ. Toulouse, CNES/CNRS/INRAE/IRD/UPS, Toulouse, France; 3 Departamento de Análisis Económico, Universidad de Zaragoza, Zaragoza, Spain; 4 Facultad de Biología, Universidad Autónoma de Madrid, Madrid, Spain; 5 Departamento de Geografía y Ordenación del Territorio-Instituto Universitario en Ciencias Ambientales de Aragón (IUCA), Universidad de Zaragoza, Zaragoza, Spain

**Keywords:** Cryospheric science, Grassland ecology

## Abstract

In mountain areas, the phenology and productivity of grassland are closely related to snow dynamics. However, the influence that snow melt timing has on grassland growing still needs further attention for a full understanding, particularly at high spatial resolution. Aiming to reduce this knowledge gap, this work exploits 1 m resolution snow depth and Normalized Difference Vegetation Index observations acquired with an Unmanned Aerial Vehicle at a sub-alpine site in the Pyrenees. During two snow seasons (2019–2020 and 2020–2021), 14 NDVI and 17 snow depth distributions were acquired over 48 ha. Despite the snow dynamics being different in the two seasons, the response of grasslands greening to snow melt-out exhibited a very similar pattern in both. The NDVI temporal evolution in areas with distinct melt-out dates reveals that sectors where the melt-out date occurs in late April or early May (optimum melt-out) reach the maximum vegetation productivity. Zones with an earlier or a later melt-out rarely reach peak NDVI values. The results obtained in this study area, suggest that knowledge about snow depth distribution is not needed to understand NDVI grassland dynamics. The analysis did not reveal a clear link between the spatial variability in snow duration and the diversity and richness of grassland communities within the study area.

## Introduction

Alpine grasslands have exceptional ecological value as they host a very high biodiversity and are the basis of livestock grazing during the summer months^1–3^. They occupy large areas in mountain regions^4^ at elevations where a significant snow cover is present during winter and spring^5^. Their growth is controlled by climate variability and snow cover dynamics^6^ as the plants must cope with a growth period limited by low temperatures and snow duration^7,8^. The high spatial variability characterizing mountain terrain causes strong variations in snow accumulation patterns over short distances^9,10^ which translate into snow depth distribution and its persistence^11–14^. This spatial variability can strongly influence the distribution and phenology of plant communities^15–17^, as well as the occurrence of endemic or very rare species with special conservation interest and thus must be included along with other meaningful variables (elevation, soil thickness, slope)^5,18^ when analyzing communities distribution and its temporal evolution^19,20^.

Important shifts in the magnitude and duration of snowpack are occurring due to ongoing climate change and are expected to continue in temperate mountains, with shorter snow seasons and a significant rise in snow line elevation^21^. These changes may have important implications for plant biomass and net above-ground production as a longer growth period may be expected^22^, but the significance of these changes is still under discussion. At the same time the distribution and abundance of plants may change as temperatures rise^23^, and, additionally, precipitation changes are highly uncertain and uneven in mountain areas^24–26^. In recent decades, important changes in plant species distribution and productivity trends have been observed in mountain areas, which have been mainly related to snow cover variations^27–30^, making the comprehension of snow–vegetation interactions highly relevant.

Since the space-borne sensor breakthrough in environmental science, several researchers have paid attention to the relationship between vegetation phenology and snow cover evolution^20,31–33^. These works have shed light on the dependence of alpine vegetation productivity on snow dynamics over large areas and how this vegetation evolves along different temporary windows^8,18,34,35^. For instance, it is known that variations in the snow-melt date control the greenness onset over extensive mountain regions^22^. However, a full comprehension of these interactions requires high and very high spatial resolution imagery^31^, for which on-site observations are needed^20^. In this regard, the combination of on-site observations with space-borne and close-range remote sensing acquisitions has demonstrated good capability for detecting short-term changes with well-established metrics for monitoring plant communities structure evolution and snow dynamics^4,33^.

In the context of remote sensing imagery, vegetation productivity increases are identified as “greening” and vegetation productivity decreases are referred to as “browning”^34,36^. In remote sensing studies over extended areas the *Normalized Difference Vegetation Index* (NDVI)^37,38^ is considered a good proxy for determining the onset of the growing season and the greenness peak, and many grassland evolution studies are based on the NDVI^28,31,34,39^, obtaining the same conclusions when equivalent indexes are used^8,40^. In this study “greening” is used to define the period with a NDVI increase. Over extended areas, the snow melt-out date is usually derived from the *Normalized Difference Snow Index* (NDSI)^41^ from satellite observations, setting a pixel as snow-free/snow-covered depending on a threshold value^42^. Other studies are based on direct snow depth and vegetation height acquisitions, but these typically require on-site observations with automatic weather stations (AWSs) measuring sensor–ground distance^6^. Several studies have investigated the relationship between the snow cover and alpine grassland productivity at high spatial resolution in numerous plant survey plots^15,16^. However, more detailed studies are needed to understand the role of micro-relief characteristics^43,44^. In this sense, Unmanned Aerial Vehicles (UAVs) enable the acquisition of detailed observations of ecological systems not achievable hitherto from satellite acquisitions, allowing the characterization of processes not monitored before^45^.

This study was designed in light of the lack of high spatial resolution studies to understand the influence that snow dynamics exert on alpine grassland growth, while also giving insight into the distribution of plant communities. The main goal is to shed light on the relation between the seasonal evolution of grassland greening and both the snow melt-out date and the snow depth observed the previous winter. The main hypothesis is that the snow disappearance date determines the onset of the grassland greening period, with a primary impact on the maximum NDVI values reached afterwards. As previous studies have noted, the snowpack can exert an influence on plant communities distribution^15,16^. Potential differences in plant species observed over short distances will also be assessed in view of the snow melt timing.

**Figures 1:**
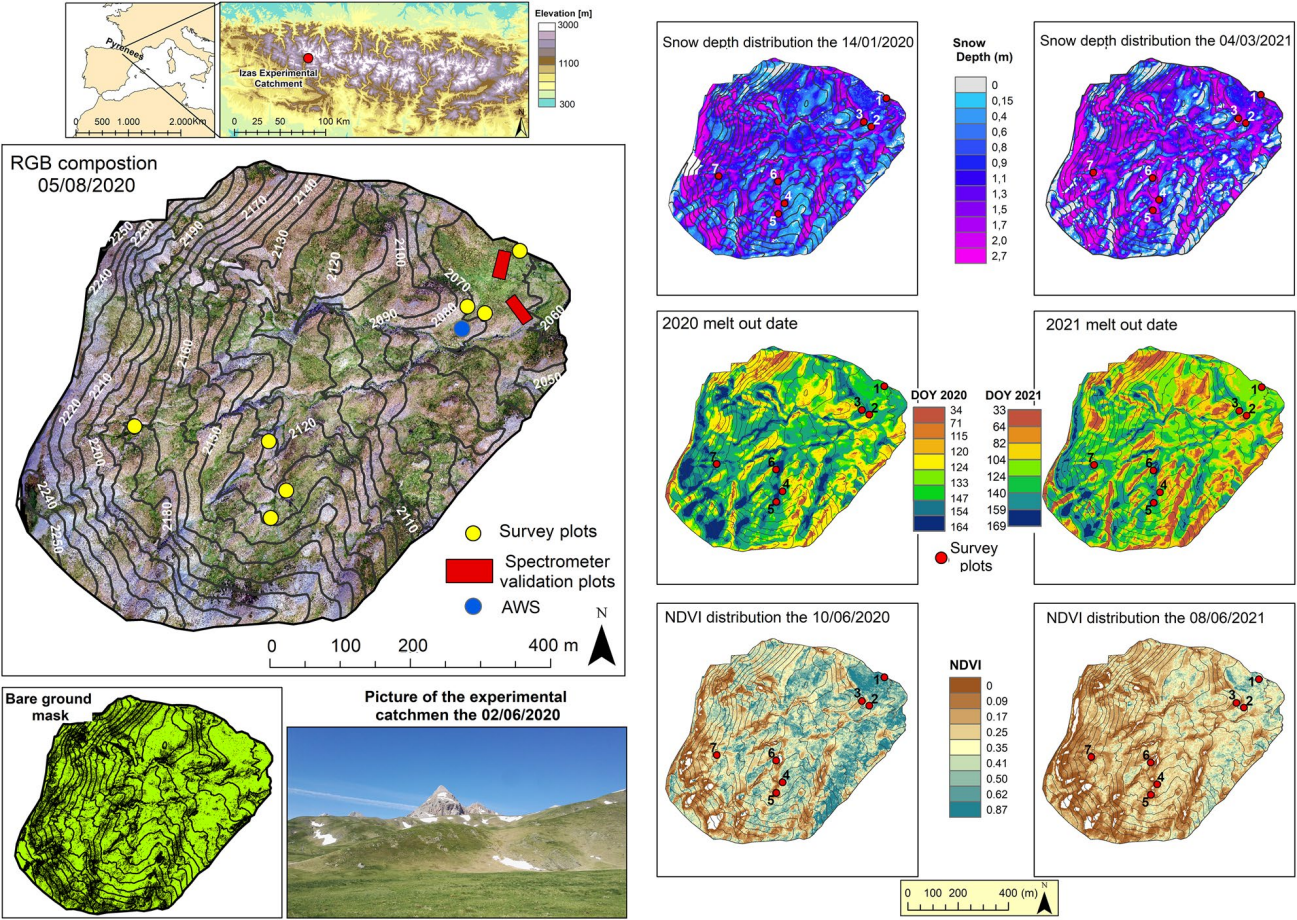
Left side panels show the location of Izas Experimental Catchment, an RGB composition obtained for the UAV flight of the 5 August 2020 including the location of plant species survey plots, the validation area of the UAV transported multispectral camera, and the location of the Automatic Weather Station (AWS). In the bottom right side the mask for bare ground and a picture showing an overview of the catchment are included. Right side panels show the distribution of different variables analyzed in this work for 2019–2020 and 2020–2021 snow seasons. The upper right maps show the snow distribution around peak snow accumulation, middle right maps depict the melt-out dates and the bottom right maps show the NDVI distribution by the end of snow melting period. The bare ground mask and an overview of the catchment obtained from the spectrometer validation plots are shown at the bottom left side. All these maps where derived from the UAV acquisitions described in the manuscript. The maps layout, the legends, the scale bars etc., of Figure were generated by J. Revuelto in ArcMAP 10.8 (https://www.esri.com/en-us/arcgis/products/arcgis-desktop/resources). The picture of the Experimental Catchment the 2 was obtained by J. Revuelto.

The methodology later detailed relies on UAV observations of the snow depth distribution and the NDVI acquired at Izas Experimental Catchment^46^, in the Central Pyrenees. This site is characterized by a long-lasting snowpack and a heterogeneous topography comprised between 2000 and 2300 m above sea level (masl). Izas Experimental Catchment (Fig. 1) is mostly covered by alpine grassland (about 85%)^47^ dominated by *Festuca eskia* and *Nardus stricta*. The high grassland cover that characterizes this site is representative of subalpine areas in the Central Pyrenees^48–50^. This fact, together with the long persistence of snow above 2000 masl in the Pyrenees^51,52^, heightens the value of the conclusions obtained in this small study area, which might be extended to other mountain areas in temperate latitudes with similar characteristics.

In the 2019–2020 and 2020–2021 snow seasons, 10 and 7 high spatial resolution (1 m × 1 m grid cell size) snow depth distribution maps covering 48 ha were derived from RGB (red, green, blue) images acquired with a fixed-wing UAV. The spatial distribution of the melt-out date was obtained from these maps. Likewise, over the snow melting period, the NDVI distribution was obtained at the same spatial resolution with a multispectral camera. The NDVI was retrieved for 9 (in 2020) and 5 (in 2021) dates between April and August. With these UAV data (see Table S1 in Supplementary material), it was possible to determine the evolution of grassland greening and relate it to snowpack variables (snow depth accumulated in previous months and melt-out date) and the temporal evolution of the meteorological conditions observed at the AWS installed in the catchment^46^. Figure 1 includes an overview of the spatial data available in the study area.

Moreover, by the end of 2021 melting season (July 2021), seven survey plots distributed across the study area were set up to study vegetation and specifically to analyze its structure (cover and floristic composition) and different diversity indices (*Shannon’s H*, *Pielou’s J*, *Simpson’s D*, *alpha*, *beta*, *soil coverage*), informations which cannot be retrieved by satellite or UAV observations. To the best of our knowledge, this is the first study exploiting very high spatial resolution observations of both snow cover and grassland greening evolution based on UAV acquisitions, and also combining them with plant diversity indexes computed in several vegetation plots.

## Results

### Main meteorological conditions over the study period

The two snow seasons analyzed had contrasting characteristics, with a short duration and shallow snowpack in 2020–2021 season and an average snow year in terms of duration and accumulation^46^ in 2019–2020 (Fig. S1 in Supplementary material). The first snow season also had milder temperatures than the 2020–2021. Precipitation in spring 2019–2020 was more frequent and intense.

### NDVI response to snow melt-out

**Figure 2:**
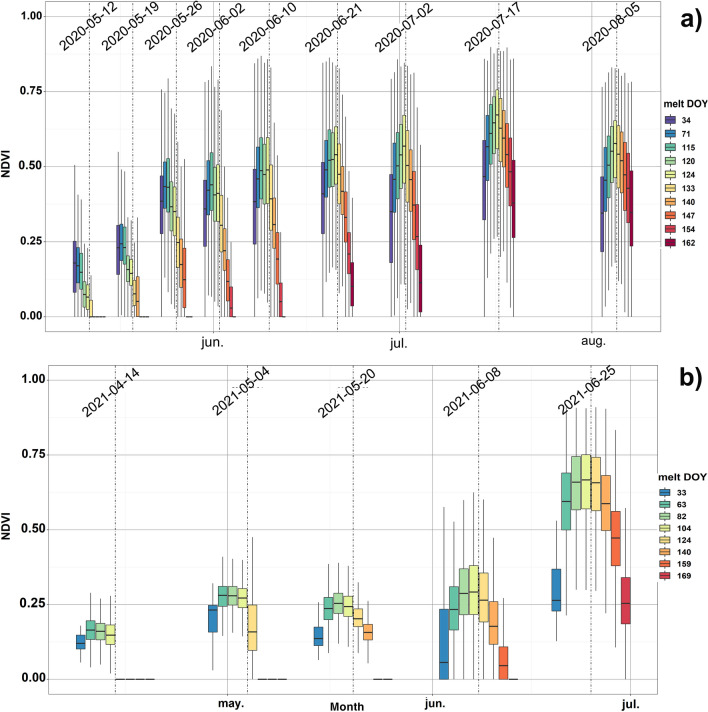
Box plots depicting the temporal evolution of the NDVI for the UAV acquisition dates categorized by the melt-out date respectively observed in 2019–2020 (**a**) and 2020–2021 (**b**). The boxes show first and third quantiles and the horizontal line inside each box the median. Whiskers of each plot include maximum and minimum values for each category and day.

The box plots in Fig. 2 show the temporal evolution of the UAV NDVI acquisitions categorized by snow melt-out date (NDVI observed in the snow-free). These box plots show that grassland greenness onset is closely related to snow disappearance date. In both snow seasons, the NDVI values start to rise in the later UAV snow observations. For the first seasonal NDVI acquisitions, some areas were snow covered and thus the NDVI was not computed inside. In the following UAV acquisitions the snow of these areas progressively melted-out and thus the NDVI gradually increased in the later observations. Similarly snow free areas in the first UAV acquisitions had overall low NDVI values (average NDVI < 0.3) increasing in the later acquisitions. The variability observed in the initial NDVI values is most likely related to the snow melt-out derived from the recursive snow depth observations. In this way, areas with the same melt-out date depict the snow extent melted between two consecutive UAV acquisitions (from 4 to 20 days among consecutive UAV flights). This hampers determination of the exact onset delay between the snow melt-out date and the greenness onset.

In the 2019–2020 snow season, the higher frequency of UAV acquisitions allows a more precise analysis of NDVI trends. The maximum NDVI values observed for the different melt-out date categories and the greenness onset after the snow disappearance show a rapid NDVI increase for areas with melt-out date around 3 May [day of year (DOY) 124] which reach peak NDVI (~ 0.7). For areas with later snow disappearance (10–21 June), pasture greening is also quite rapid, but the maximum values reached are smaller (~ 0.6). The last UAV observation of the NDVI in 2020 showed the early phase of grassland senescent, as NDVI decreased for the entire study area. Despite the lack of more frequent acquisitions and the lack of UAV observations during the 2021 summer, the second snow season shows similar behavior of the NDVI evolution (onset, maximum NDVI and tendency) to that observed in the previous season. In view of the contrasting snow depth evolution and the differences in temperature and precipitation observed in 2019–2020 and 2020–2021 (Fig. S1 in Supplementary material), it is remarkable that, in both seasons, the spatial maximum NDVIs are reached in areas with melt-out date occurring between late April and early May.

Overall, areas with the earlier melt-out date do not reach NDVI values as high as those observed in zones where snow disappearance occurs later. This is particularly noticeable for areas where the melt-out date occurs before April (DOY < 105), which have mean NDVI values below 0.65 at peak productivity (observed in July). Similarly, those zones with the latest snow melting do not overtake the maximum mean NDVI values (0.7), reached in areas with melt-out date around DOY 105–124 (15 April–4 May). Interestingly, this NDVI evolution is observed in both snow seasons, illustrating that there is an optimal melt-out date to reach maximum NDVI values. This outcome suggests that the study area can be classified into three main groups in view of the melt-out date: early snow melt-out (DOY < 105), mid snow melt-out (or optimum melt-out, 105 < DOY < 125), and late snow melt-out (DOY > 125). In 2020 the extent covered by these classes is: 30% for early snow melt-out, 22% for mid melt-out and 48% for late melt-out; whereas in 2021 these are respectively 40%, 37% and 23%.

### NDVI response to snow depth

**Figure 3:**
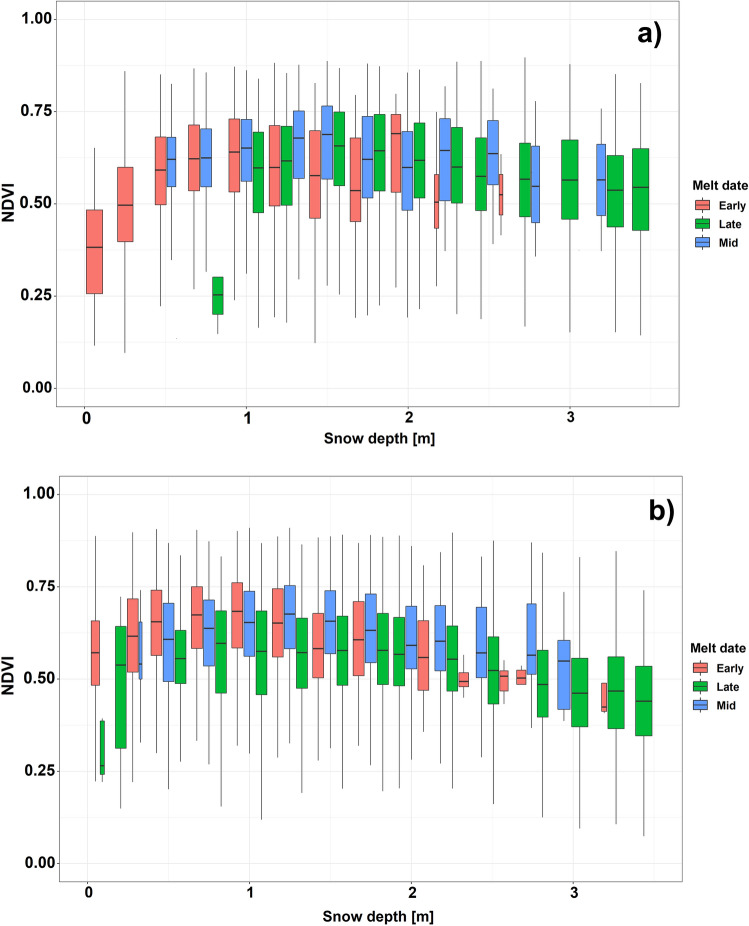
Box plots of maximum snow depth and maximum NDVI values observed in 2020 (**a**) and 2021 (**b**). Values have been categorized in the three main melt-out date groups (early melt, mid melt and late melt) and grouped by 0.25 m snow depth intervals. The boxes show first and third quantiles and the horizontal line inside each box the median. Whiskers of each plot include maximum and minimum values for each category.

The box plots in Fig. 3 show the comparison between the maximum NDVI and the maximum snow depth observed in each snow season (seasonal maximum snow depth on each pixel), grouped in 0.25 m snow depth intervals and categorized in the three major melt-out date categories (early, mid and late melt-out DOY). A clear tendency of maximum NDVI values when compared to the maximum snow depths is not observed. The three melt-out date groups exhibit high dispersion for peak NDVI, suggesting that peak snow accumulation (observed between late March and early April) is not related to peak grassland productivity. Only for high snow accumulations (> 2 m) exist a slightly negative tendency between snow depth and NDVI, which is explained by a later melt out date in those locations, tending to prevent high NDVI values. Figure 3 also demonstrates that maximum snow depth cannot be used to derive peak NDVI, as both high and low peak snow depths can later have low NDVI values and thus. Similarly, the correlation between NDVI and snow depth (not shown) was only slightly negative (albeit statistically significant) between observations having an intervening temporal gap (the NDVI and snow depth observations) of two to three weeks. For late NDVI observations (July/August), correlations with previous snow depth observations were close to zero (not shown), showing that the maximum NDVI is not related to the snow depth distribution observed some months before.

### Plant diversity indices; NDVI and topographic characteristics of the plots

**Figure 4:**
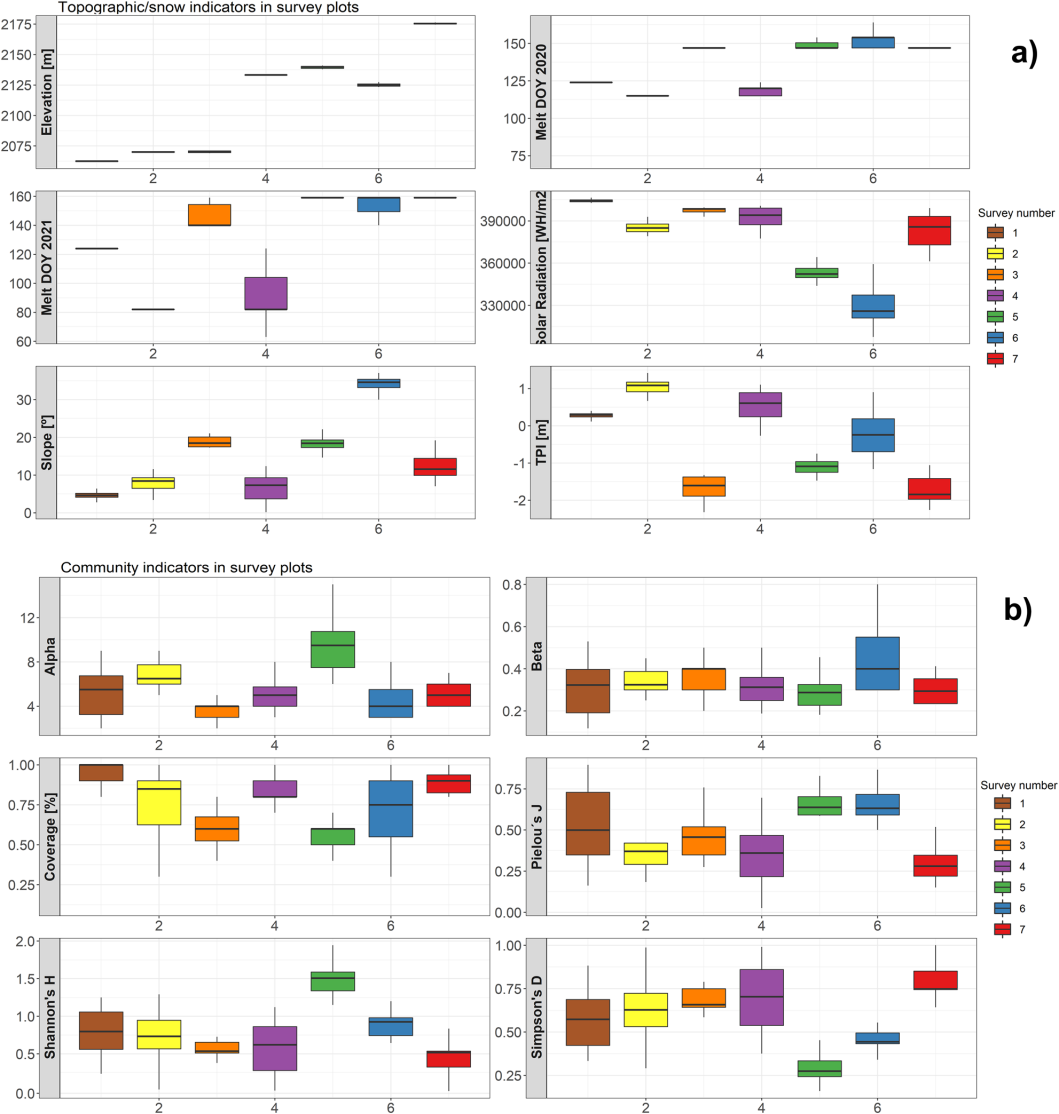
Box plots showing the dispersion of the topographic variables (**a**) and grassland communities’ indexes (**b**) observed at the survey plot locations. The boxes show first and third quantiles and the horizontal line inside each box the median). Whiskers include maximum and minimum values for each variable observed in the plots.

A wide variety of grassland species was observed in the seven survey plots (Supplementary material Table S2); however, in all plots the dominant species is *Festuka eskia*, which shows high and very high plant coverage in most subplots (see plots 4 and 5 in Table S2). Two other species are also present in all plots (Table S2), *Pilosella officinarum* and *Nardus stricta*. The other species observed had smaller plant coverage, with a wide variety of plant composition between the plots. The frequency distribution derived from the plant cover of all species yields a wide variety of indexes describing plant communities. The average species richness (*alpha*) and the plot’s species turnover (*beta*) denote that plot 5 had the widest variety of plant species (Fig. 4), while plot 6 had fewest. The other plots had similar species richness. Overall plant cover in the plots was high, and, except for plot 5, which had subplots showing coverage between 50 and 70%, the coverage in all of them ranged between 75 and 100%. *Shannon’s H* depicts the marked variety of species observed in plot 5, whereas the species evenness (*Pielou’s J*) in subplots 5 and 6 is higher than that observed in the other five plots. Plots 4 and 7 exhibit the highest *Simpson’s D* average values, denoting the dominance of a few plant species, whereas in plots 5 and 6 a wider diversity is observed.

Despite the variety in plant species and in plant diversity indices, there was no evident relationship between plots’ topographic characteristics and their snow melt-out dates. The topographic characteristics alone (Fig. 4) do not explain the dispersion in plant diversity indices. The slopes of the surveyed plots show that plant coverage is higher for flatter plot locations. Similarly, the higher solar radiation is, the lower *Shannon’s H* is. This indicates that, in this study, areas with enhanced solar radiation have fewer species varieties and are dominated to a greater extent by *Festuka eskia*. The melt-out dates, which span about 45 days in 2020 and more than 80 in 2021, do not show any notable relationship to plant diversity indexes. However, the temporal evolution of the NDVI in both years is closely related to the melt-out date (Fig. S2 in Supplementary Material), in agreement with the NDVI patterns observed for the entire catchment (Fig. 2).

## Discussion

This work aimed to shed light on the understanding of snowpack influence on grassland greening in mountain areas over short distances. From previous works in snow-dominated areas^8,22,34,35^ this hypothesis was established: the snow disappearance date determines the onset of the grassland greening period, with a primary impact on the maximum NDVI values reached later. The results shown here confirm this hypothesis, demonstrating, with very high spatial resolution observations, the major impact that snow melt-out date has on the temporal evolution of alpine grassland greening. The frequent snow-depth and NDVI observations at Izas Experimental Catchment have also made it possible to confront a question recently raised when investigating shrub growth in snow-dominated areas^44^: “(…) *earlier snow melt-out on alpine shrub growth: the sooner the better?*”. The UAV observations presented here demonstrate that, for alpine grasslands, sooner is not always better^53,54^. Moreover, these acquisitions show that the optimal productivity, in terms of maximum NDVI, is found in areas where melt occurs at intermediate dates (late April–early May, termed mid melt-out or optimum melt-out). Nevertheless, there might be other processes or variables (soil depth and characteristics, terrain curvature, aspect or solar exposure…) contributing to reach peak NDVI values as the dispersion on the NDVI values observed in this study demonstrate. Zones where the melt-out date occurs before (early melt-out) or later than (late melt-out) the optimum melting period show lower maximum NDVI values and thus, it is expected, lesser vegetation productivity. This outcome is significant because many works have already highlighted the major role of snow melt dynamics in grassland greening dynamics^8,22,34,35,55,56^ but fewer emphasize the decrease in grassland productivity if the snow disappearance occurs too soon^57–59^ or too late. The high spatial resolution of the observations has proven that grassland greening evolution can be highly variable over (very) short distances and it allows a classification in terms of the snow disappearance date as: early snow melt-out, mid snow melt-out, and late snow melt-out.

The discretization of Izas Experimental Catchment on a wide variety of melt-out dates also reveals two greenness trends: one with a moderate NDVI temporal increase which does not reach maximum NDVI values and is observed for early snow-melt areas, and the other with a rapid NDVI increase which can reach maximum NDVI values (for mid melt-out) or not (for late melt-out) but both with a similar temporal tendency. Some works observed that an earlier snow melt implies more productive grassland^55^, but certainly there are other meteorological constraints affecting grassland dynamics. Thus, if snow disappears too soon, the plants are exposed to spring frost which does not enable a longer growing season^57^ and thus restricts plant development^60^. Areas with the earliest snow melt match the regions with shallower snowpack^10^ at times of peak snow accumulation. These are typically observed in mountain ridges^61,62^, and frequently have less developed and shallow soils^63^. In Izas Experimental Catchment, the shallowest snowpack accumulations at peak accumulation time^14^ match areas with very limited soil presence^64^ and were masked in the analysis (Fig. 1) because no plants are present here. In these areas, the wind erosion of the snowpack prevents the establishment of a long-lasting snowpack that might protect soil from extreme temperatures. In turn, this affects the soil nutrient cycle, and thus grassland growth and its productivity is also drastically reduced^65^.

The long period covered by the NDVI acquisitions obtained in 2020 allowed determination of the maximum greening, as the last UAV flight in August 2020 already showed a marked NDVI decrease throughout the study area. The impact of snow dynamics in the senescing part is known to be minor as other meteorological variables control this period^8,19,39^, so analogous behavior was expected in 2021 (not observed due to the UAV crashing in July 2021). The grassland greening evolution was observed in two snow seasons with marked snow and climatic differences. Thus, the similar results obtained in both study years allow the conclusions of this work to be extended to years with climatic dissimilarities. Nonetheless, to extract more robust conclusions and catch the high temporal variability that characterize the snowpack accumulation in the Pyrenees it is encouraged to maintain similar observations in this mountain range^66,67^. Similarly, the use of UAV to retrieve high spatial resolution observations has constrains as the limited spatial extent of the study area. In this regard, UAV studies in mountain areas hardly exceed 100 ha^68–70^. This work is based on observations obtained on a small study area where the findings are valid. These might be extended to areas of similar characteristics with equivalent plant coverage and diversity, but would require additional observations to validate them if extended to larger areas.

Another interesting outcome of this research is that the snow depth distribution observed at peak snow accumulation does not explain the peak NDVI distribution. Small correlations between the snow depth and the NDVI were only observed in acquisitions close in time (about 2–3 weeks difference between the snow depth and the later NDVI observation) likely due to the coming snow disappearance in areas with shallow snow depths. This result strongly suggests that NDVI dynamics can be studied in the light of the spatial distribution of the snow melt-out date. The latter variable can nowadays be retrieved for large areas from a wide variety of high-resolution optical satellite sensors (Sentinel, SPOT, MODIS and others). However, a UAV still provides higher spatial and also higher acquisition flexibility than satellite sensors. This is clearly beneficial in areas where clouds limit satellite observations, as a UAV may permit acquisition at the exact times when skies are clear.

Although soil characteristics, humidity content and other variables with notable implications for grassland communities distribution^71,72^ have not been investigated here, the major role played by snow melt dynamics in grassland greening evolution has been shown. Other works have found remarkable differences in alpine grassland communities related to snow melt timing^15,73^, but within the seven survey plots established in the Izas Experimental Catchment, no clear relationship between plant communities and snow dynamics or topographic factors was found. The only notable outcome of plant community surveys is that plots with higher solar radiation have less species richness while flatter plots have higher plant cover, which might be connected with the thicker soils usually found in these areas. This demonstrates that the snowpack is not the unique variable explaining plant communities: although it might have an important role, it must be considered together with other topographic and soil variables^5,19^. The noteworthy presence in all the plant communities sampled of *Festuca eskia*, *Nardus stricta* and, to a lesser extent, *Festuca nigrescens*, is decisive and relegates the contribution of the rest of the species to the relationship of vegetation greening with the NDVI, but allows a spatial comparison of NDVI evolution. It should be noted that in many plant communities the predominance of biomass is accounted for by a few species (especially of genus *Festuca*, *Carex*, *Trifolium*)^5^, which would allow simplifying the greenish to NDVI ratio estimation by limiting it, in a first approximation, to these species. Moreover, these species dominate plant communities in large alpine and sub-alpine areas of the Pyrenees^49^, what might enable to apply similar analysis to other areas of this mountain range. The short growing season on the alpine floor, is a strong constraint conditioning plants to fast development (growth, flowering and fruiting) after snowmelt in order to complete as soon as possible their life cycle, regardless of their taxonomy, morphology, life form and reproductive biology. This fact suggests a similar temporal behavior towards the greenish of most of the flora.

In view of the expected changes in mountain areas in the coming decades, the snow cover is likely to decrease in both extent and duration^21^. These shifts in spatio-temporal evolution of the snowpack will impact grassland greening dynamics^30,36^ but will also, if maintained over time, influence plant communities distribution^74^. In some locations, these changes might benefit species migrations from lower to higher elevations, but in other locations earlier snow melt-out in the future could impede the establishment of some species as these might not be protected from atmospheric extremes.

## Conclusions

The results of this work have shown the important role that the snow melt-out date plays in the NDVI evolution in alpine grassland. Peak NDVI values are reached in areas with a melt-out date around late April or early May. Conversely, those zones showing an earlier snow disappearance do not reach NDVI values as high as those obtained in areas of mid melt-out. Similarly, areas with a later snow disappearance do not achieve peak NDVI values. This behavior suggests that there is an optimum melt-out date to reach maximum NDVI values, and that grassland greening is penalized by snow melting that occurs too early or too late. In Izas Experimental Catchment no major differences in grassland communities were related to snow dynamics or NDVI evolution. Nonetheless, there is a predominant species in all plots (*Festuka eskia*) which may have hampered the observation of potential differences in the NDVI evolution of the survey plots. Results also revealed the negligible relationship between maximum NDVI and peak snow depth values, but also the minor contribution made by previous snow depth observations to explaining NDVI. Hence, grassland dynamics studies on mountain areas might only require snow melt-out information, which is easily retrieved from binary snow cover maps from spaceborne, airborne and on-site sensors. Further work for longer periods, over extended mountain areas, and covering a higher altitudinal gradient, is recommended to fully understand grassland dynamics in snow-dominated areas and validate the conclusions obtained here in a small study area.

## Methods

### UAV-based NDVI and snow depth observations

The high spatial resolution database of both snow depth and NDVI distribution was retrieved in Izas Experimental Catchment by means of a fixed-wing UAV, a *SenseFly eBee-Plus*. Either a RGB camera (*S.O.D.A.*) or a multispectral camera (*Parrot Sequoia*) was mounted on the UAV and acquired images of the study area during the UAV flights. These images were then processed in *Pix4Dmapper* (version 4.4.12), which is a Structure from Motion (SfM) software that generates orthophotos and 3D point clouds representing the snow/ground surface. The NDVI maps were computed using the red and near-infrared bands^75,76^ of the multispectral images. The snow depth was computed by comparing the 3D point clouds with snow presence and a point cloud generated from images retrieved without snow. Following a detailed acquisition protocol and under good illumination conditions, the accuracy of the UAV snow depth observations in this study area is below 0.19 m, as previously demonstrated in Izas Experimental Catchment^68^. By contrast, the UAV-based NDVI observations were not previously evaluated in this study area and, although a thorough methodology was followed, an error assessment for the product generated was needed^77^.

The orthophotos derived from the *Parrot Sequoia* multispectral camera bands and the NDVI for the UAV flight of 5 August 2020 in different sectors of the study area (Fig. 1) were compared to observations retrieved with an Analytic Spectral Devices spectrometer (ASD FieldSpec4 SR), with a bare fore optic (25°). ASD reflectance was calibrated using a white Spectralon panel (Labsphere Inc., North Sutton, NH, USA)*.* Several transects (5 on each validation plot) were established within the validation plots to retrieve the grassland radiometric signal (50 points on each transect). ASD reflectance values were convolved to the spectral response functions of the Sequoia camera for comparison. This comparison showed an RMSE in the NDVI of 0.047 and a linear adjustment between both observation devices (camera Sequoia and ASD spectrometer) with an R^2^ = 0.84 (see Fig. S3 in Supplementary material). These values demonstrate the reliability of the NDVI acquisitions of the UAV exploited here. From the same UAV flight, the bare ground mask was derived based on a threshold value in the blue band of the RGB orthophoto.

The melt-out date computation within the catchment was obtained from the snow depth maps. Those sectors of the study area having snow presence in one UAV acquisition but not in the subsequent flights were set with a melt-out date equal to the last day with snow. This choice was made to ensure an accurate distribution of the last date with snow presence observed within the study area. Thus it would be more accurate to name this variable as “last snow occurrence date”, but for simplicity we named it melt-out date.

### Plant communities indexes in survey plots

The vegetation was sampled in late June 2021. Based on the digital terrain model, seven zones of about 0.1 ha were selected in the study area (Fig. 1), stratified according to their topographic characteristics (elevation, exposure, slope and terrain curvature), which, in turn, determines the snow lifespan and the growing season. Inside each plot, two 10 m perpendicular transects were marked. Along each of these lines ten squares of 0.5 × 0.5 m (subplots), separated by 1 m and placed alternately on both sides of the line, were delimited to record plant cover including that of each plant present within the plot. Inside each subplot all plant species were computed and their coverage determined based on the line-point intercept method^78^. The data obtained from these surveys served to determine species richness and plant coverage, later exploited to determine plant community cover and plant diversity indexes (*Shannon’s H, Pielou’s J*, *Simpson’s D*, alpha, beta, soil coverage)^79,80^.

## Supplementary Information

Supplementary Information.

## Data Availability

The database of UAV acquisition obtained in Izas Experimental Catchment will be made freely available in Zenodo repository after publication with this https://doi.org/10.5281/zenodo.7038071.
