# Supplementary material for: Intermediate snowpack melt-out dates guarantee the highest seasonal grasslands greening in the Pyrenees
Source: Sci Rep. 2022 Oct 31;12:18328. doi: 10.1038/s41598-022-22391-x (PMC9622740; doi:10.1038/s41598-022-22391-x)
Supplement: Supplementary file 1 — Supplementary Information. [file 41598_2022_22391_MOESM1_ESM.pdf]

Intermediate snowpack melt-out dates guarantee the highest seasonal grasslands greening in the Pyrenees

J. Revuelto[1], D. Gómez[1], E. Alonso-González[2], I. Vidaller[1], F. Rojas-Heredia[1], C. Deschamps-Berger, J. García-Jiménez[1], G. Rodríguez-López[3], J. Sobrino, R. Montorio[4], F. Perez-Cabello[4], J.I. López-Moreno[1]

[1] Instituto Pirenaico de Ecología, Consejo Superior de Investigaciones Científicas (IPE-CSIC), Zaragoza, Spain
[2] Centre d'Etudes Spatiales de la Biosphère, CESBIO, Univ. Toulouse, CNES/CNRS/INRAE/IRD/UPS, Toulouse, France
[3] Departamento de Análisis Económico. Universidad de Zaragoza.
[4] Departamento de Geografía y Ordenación del Territorio-Instituto Universitario en Ciencias Ambientales de Aragón (IUCA), Universidad de Zaragoza. Zaragoza, Spain.
Corresponding author: jrevuelto@ipe.csic.es

Supplementary material:

Year	Date	DOY	SnowDepth Obs.	NDVI Obs.
2020	February 3	34	X	
	February 24	55	X	
	March 11	71	X	
	April 24	115	X	
	April 29	120	X	
	May 5	124	X	
	May 12	133	X	X
	May 19	140	X	X
	May 26	147	X	X
	June 2	154	X	X
	June 10	162		X
	June 21	173		X
	July 2	184		X
	July 17	199		X
	August 5	218		X
2021	February 2	33	X	
	March 4	63	X	
	March 23	82	X	
	April 14	104	X	X
	May 4	124	X	X
	May 20	140	X	X
	June 8	159	X	X
	July 25	169		X

Table S1: Summary of UAV acquisition during the study period. DOY: Julian Day of the Year.

**1**	2	3	4	5	6	7
Athoxatum odoratum	*Campanula scheuchzeri*	*Festuca eskia*	*Festuca eskia*	*Alchemilla saxatilis*	*Bellardiochloa variegata*	*Androsace laggeri*
Carex nigra	*Cardamine bellidifolia*	*Jasione laevis*	*Nardus stricta*	*Androsace laggeri*	*Campanula scheuchzeri*	*Campanula scheuchzeri*
Cerastium fontanum	*Festuca eskia*	*Leontodon hispidus*	*Pilosella officinarum*	*Androsace vitaliana*	*Crocus autumnalis*	*Conopodium sp.*
Conopodium sp.	*Festuca nigrescens*	*Leontodon pyrenaicus*	*Plantago alpina*	*Anthyllis vulneraria*	*Festuca eskia*	*Festuca eskia*
Dactylorhiza maculata	*Jasione laevis*	*Meum athamanticum*	*Jasione laevis*	*Botrychium lunaria*	*Festuca nigrescens*	*Festuca nigrescens*
Festuca eskia	*Leontodon pyrenaicus*	*Nardus stricta*	*Liquen*	*Campanula scheuchzeri*	*Nardus stricta*	*Galium cometerhizon*
Festuca nigrescens	*Liquen*	*Pilosella officinarum*	*Campanula scheuchzeri*	*Conopodium sp*	*Pilosella officinarum*	*Jasione laevis*
Jasione laevis	*Luzula spicata*	*Plantago alpina*	*Leontodon pyrenaicus*	*Euphrasia alpina*	*Ranunculus pyrenaeus*	*Leontodon hispidus*
Nardus stricta	*Musgo*	*Sedum anglicum*	*Ranunculus pyrenaeus*	*Festuca eskia*	*Trifolium alpinum*	*Leontodon pyrenaicus*
Phleum alpinum	*Nardus stricta*	*sedum brevifolium*	*Silene rupestris*	*Festuca nigrescens*	*Trifolium thalii*	*Nardus stricta*
Pilosella officinarum	*Pilosella officinarum*		*Antennaria dioica*	*Gentiana verna*		*Phleum alpinum*
Plantago alpina	*Plantago alpina*		*Sedum anglicum*	*Homogyne alpina*		*Pilosella officinarum*
Ranunculus pyrenaeus	*Ranunculus pyrenaeus*		*musgo*	*Jasione laevis*		*Plantago alpina*
Thymus praecox	*Sedum anglicum*		*Euphrasia alpina*	*Leontodon hispidus*		*sedum brevifolium*
Trifolium alpinum	*Silene rupestris*		*Vaccinium myrtillus*	*Leontodon pyrenaicus*		*Thymus praecox*
Trifolium pratense	*Thymus praecox*		*Phleum alpinum*	*Lotus alpinus*		*Trifolium alpinum*
Veronica serpyllifolia	*Trifolium alpinum*			*Nardus stricta*		*Vaccinium myrtillus*
	Vaccinium myrtillus			*Pilosella hypeurya*		
	Vaccinium uliginosum			*Pilosella officinarum*		
	Veronica fruticans			*Plantago alpina*		
				Potentilla brauniana		
				Potentilla frigida		
				Sagina saginoides		
				sedum brevifolium		
				Sibbaldia procumbens		
				Taraxacum pyrenaicum		
				Thymus praecox		
				Trifolium alpinum		
				Trifolium pratense		
				Trifolium thalii		
				Vaccinium uliginosum		
				Veronica nummularia		

Table S2: Plant species observed on each survey plot.

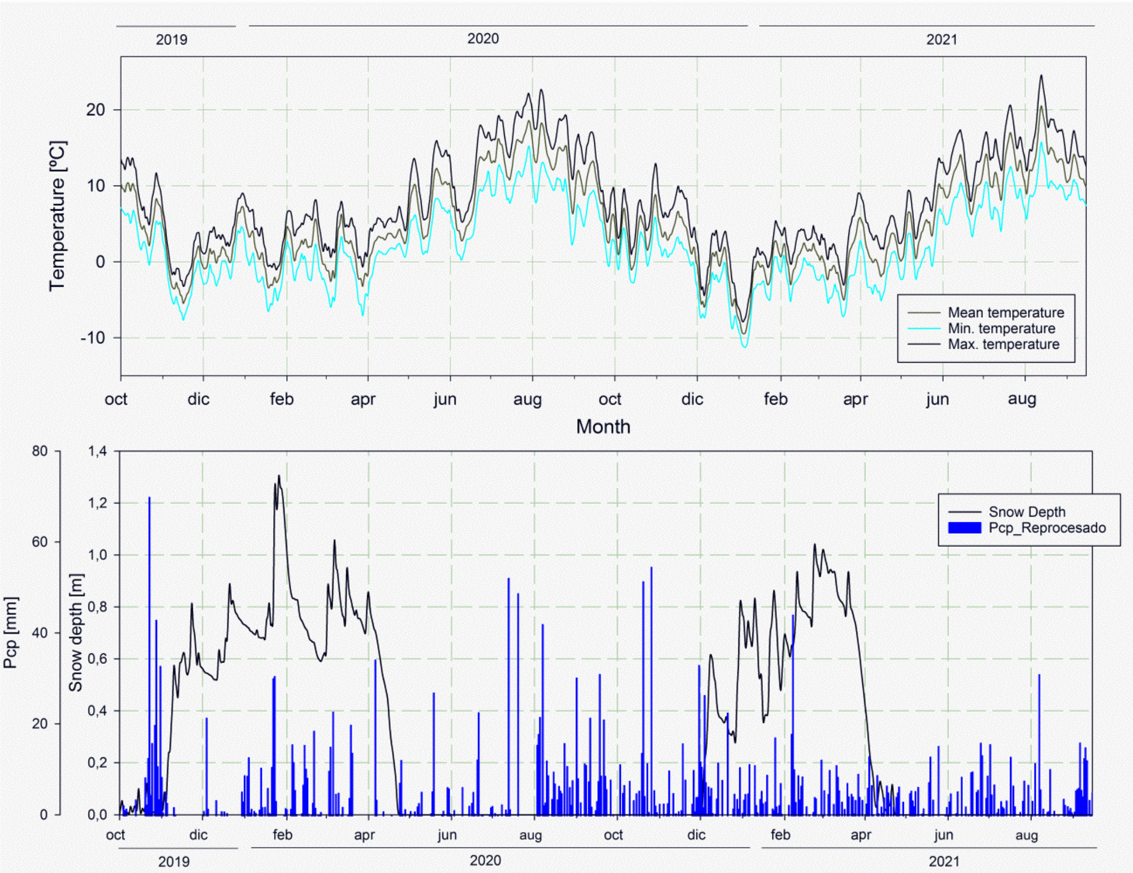

Figure S1: Upper panel shows, mean, minimum and maximum daily temperatures (5-day moving average). The lower panel depicts the temporal evolution of the snow depth and the total precipitation observed at the automatic weather station.

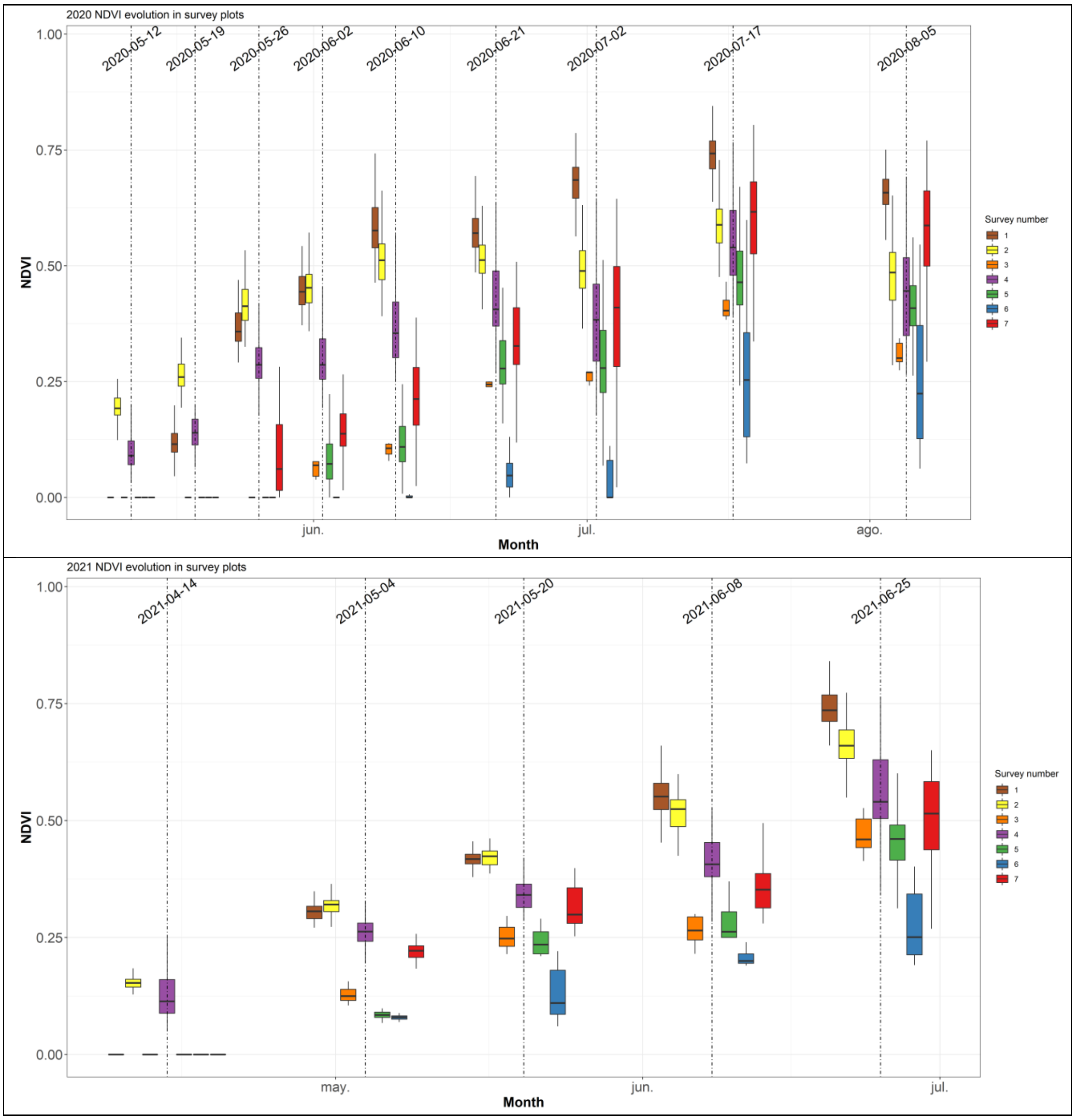

Figure S2: Box plots depicting the temporal evolution of the NDVI for the UAV acquisition dates in the plants survey plots in 2019-2020 (upper panel) and 2020-2021 (bottom panel). The boxes show first and third quantiles and the horizontal line inside each box the second percentile (median). Whiskers of each plot include maximum and minimum values for each plot and day.

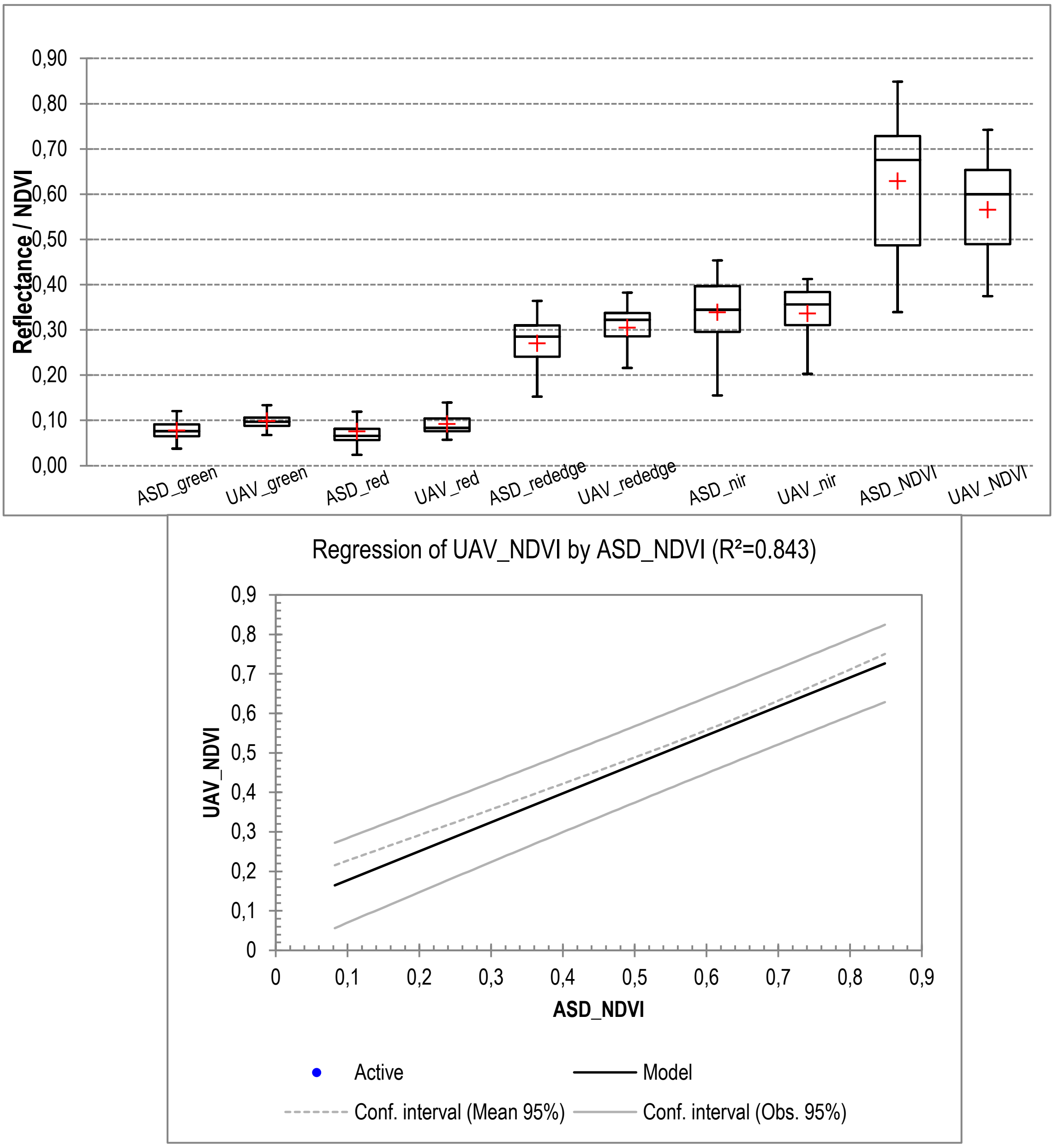

Figure S3: Box plot (upper panel) of ASD spectrometer and Sequoia camera mounted on the UAV for the four bands and the NDVI computed from the near infrared and red bands. Linear adjustment (bottom panel) between the NDVI observations of the UAV and the ASD.
